# Histologic Evaluation of Early Papilla Healing after Augmentation with Injectable Hyaluronic Acid—A Proof of Concept

**DOI:** 10.3390/jcm13144102

**Published:** 2024-07-13

**Authors:** Octavia Carolina Vela, Marius Ion Boariu, Vincenzo Iorio-Siciliano, Adrian Vaduva, Alla Belova, Stefan-Ioan Stratul, Darian Rusu

**Affiliations:** 1 Department of Periodontology, Faculty of Dental Medicine, Anton Sculean Research Center for Periodontal and Peri-Implant Diseases, Victor Babes University of Medicine and Pharmacy Timisoara, 300041 Timisoara, Romania; vela.octavia@umft.ro (O.C.V.); alla.belova@umft.ro (A.B.); stratul.stefan@umft.ro (S.-I.S.); rusu.darian@umft.ro (D.R.); 2 Department of Endodontics, Faculty of Dental Medicine, TADERP Research Center, Victor Babes University of Medicine and Pharmacy Timisoara, 300041 Timisoara, Romania; 3 Department of Periodontology, School of Dental Medicine, University of Naples Federico II, 80138 Naples, Italy; enzois@libero.it; 4 Department of Pathology, Faculty of Medicine, ANAPATMOL Research Center, Victor Babes University of Medicine and Pharmacy Timisoara, 300041 Timisoara, Romania; vaduva.adrian@umft.ro

**Keywords:** hyaluronic acid, histologic analysis, papilla augmentation, early wound healing

## Abstract

**Objectives:** This human histological study’s purpose was to histologically evaluate papillae’s healing after hyaluronic acid (HA) gel augmentation at three healing time points after one injection with hyaDENT BG^®^. **Methods:** Fifteen papillae from two patients with stage III, grade B periodontitis have been selected for this study. Every week for three weeks, five papillae were injected once with HA gel, and during the fourth week, the papillae were surgically removed as part of step 3 of the periodontal treatment. The histological analysis was performed on fifteen papillae, with five papillae corresponding to every timepoint of healing (weeks 1, 2, and 3). The primary outcome was considered to be the newly formed collagen fibers. The presence of residual HA, the integrity of epithelium or the presence of erosions/ulcerations, the presence and characteristics of inflammatory infiltrate, the presence of granulomatous reactions, and interstitial edema were considered to be secondary outcomes. **Results:** From the first to the third week, newly formed connective tissue begins to appear, while the observed HA pools (vesicles) content decreases. The density of inflammatory infiltrate was higher in the first week after injection, decreasing considerably by week 3; however, it was still visible throughout the healing time points. A granulomatous reaction was present in only three samples, while no signs of ulceration or necrosis could be observed; however, epithelial erosions could be observed on some samples after the first week. **Conclusions:** Papila augmentation with hyaluronic acid promotes new collagen formation from the second week of healing despite some foreign body granulomatous reactions.

## 1. Introduction

Patients with periodontal disease are the most susceptible to developing “black triangles” due to the loss of interdental papillae [1,2]. The aesthetics of anterior teeth are a critical component of contemporary dentistry [3], and different methods have been used to address any potential deficiencies in this area [4,5].

Although “white aesthetics” have been successfully managed through various techniques, “pink aesthetics” are still a challenge for clinicians, especially in the interdental area [6]. Following caries and improper crown margins, papilla deficiencies are regarded as the third most important aesthetic concern [7]. “Black triangles” appear when a papilla deficiency is caused by the greater distance between the contact point and the alveolar bone crest [8]. Age, periodontal disease and other diseases [9,10], the shape of the crow, and the trajectory of the cemento–enamel junction are among the factors that induce papilla recession [8,11,12]. One-third of all adults are affected by “black triangles”; however, they occur more frequently in adult patients affected by periodontal disease [13]. One of the ultimate objectives of periodontal therapy is the regeneration of the lost periodontal support tissues [14]. The interdental papilla has a restricted ability to regenerate compared to other areas of the gingiva [6]. Moreover, it has a higher susceptibility to gingival overgrowth, suggesting that it has distinct cellular and molecular properties compared to other parts of the gingiva [15].

Because of its multifactorial etiology, managing a case of papilla loss requires an individual plan based on each patient’s characteristics. There are different approaches to treating lost papillae. Correction of traumatic oral hygiene procedures is a component of the preventive strategy. Since this is a critical preventive measure against papilla loss, patients should be meticulously instructed and monitored regarding their hygiene routine [16,17]. The orthodontic approach includes closure of the interdental space. By closing the diastema and creating a contact point between the adjacent teeth, the interproximal gingival tissue will fill the space by “creeping” of the tissues [18,19]. When the biological width and the supracrestal attachment of collagen fibers are respected, prosthetic restorations can aid in the creation of adequate contacts in cases involving an abnormal tooth shape or missing teeth, potentially leading to the formation of interdental papillae [20]. Surgical approaches include different methods for papilla reconstruction. Techniques of pedicle flaps, semi-lunar coronally repositioned flaps [21], and regenerative procedures with connective tissue and bone grafts, often combined with a micro-surgical approach, are used [22,23,24]. Nevertheless, a reliable method for restoring lost papillae remains difficult to find. A significant factor contributing to this issue is the restricted blood circulation in the papilla, which poses a challenge for any type of grafting procedure. The majority of surgical methods are technically challenging, with inconsistent regeneration of the papillae, and fail to deliver successful long-term results.

As a result, different noninvasive methods have been developed to regenerate lost papillae [25,26], as well as the injection of a variety of fillers, including hyaluronic acid (HA) [27,28]. Hyaluronic acid has been used as a filler to increase the volume in other body parts such as the nose, chin, and cheeks, and also to improve the overall aspect of the face (age-related wrinkles) [29,30]. Recent research has proposed the injection of HA into connective tissue as a novel method of addressing the issue of interdental gingiva recession through the migration of fibroblasts and fibrogenesis [27,31,32]. While the involvement of HA in various biological processes during tissue repair and regeneration is well established, the specific mechanisms by which it contributes to oral soft tissue wound healing are still unknown and require further investigation [33]. A recent study using rat models examined the potential positive impact of HA film on the healing and regeneration of epithelial tissues and concluded that HA could have a beneficial effect on the healing rates of oral wounds [34]. A recent human histologic study examined the potential positive impacts of hyaluronic acid on the healing process of open-flap surgeries in the oral cavity [35]. HA was applied or not applied to the releasing incisions, using a split-mouth design, prior to suturing; a biopsy was obtained after 24 h; and clinical parameters were evaluated after 1 week. The study found that, clinically, after 1 week, HA appears to improve the remodeling of the extracellular matrix and the maturation of collagen, which may contribute to the early healing of gingival tissues and improve clinical parameters. However, HA gel does not affect the growth of new blood vessels during the initial phase of wound healing [35].

HA is a linear polysaccharide that is found in the extracellular matrix of various tissues, including connective tissue and synovial fluid [36,37]. The stability of HA preparations that are employed as fillers must be maintained over time [38,39]. These substances are derived from bacteria or animals and their therapeutic effect usually persists for a period of 6 to 12 months [40]. HyaDENT BG^®^ is a medical device consisting of a sterile gel of cross-linked hyaluronic acid that has been highly purified (class III), containing complexes of butanediol diglycidyl ether (BDDE)-crosslinked 1000 kDa-HA monomers and a native non-cross-linked HA with MW of 2500 kD [36,41].

Currently, there have been limited investigations on the impact of using HA injections as a filler to restore lost interdental papilla. Furthermore, most of the previous studies were conducted as case series with a relatively small number of participants. The technique was initially documented by Becker et al. [27] in a pilot investigation. Subsequent research produced a number of different reports with varying success rates and variable outcomes that are hard to compare because individual treatment sessions varied and there were no suitable control sites [28,31,42,43]. A small number of studies created repeatable in vivo rat models to confirm the histological and morphological alterations of the HA-augmented interdental papilla [32,44,45]. As far as we know, there are no histological evaluations of healing after papilla augmentation in humans. This study aims to compare, from a histologic point of view and at different time points, the early healing of interdental papilla reduced by periodontal disease, after augmentation with injected HA gel.

## 2. Materials and Methods

### 2.1. Study Design

**Figure 1:**
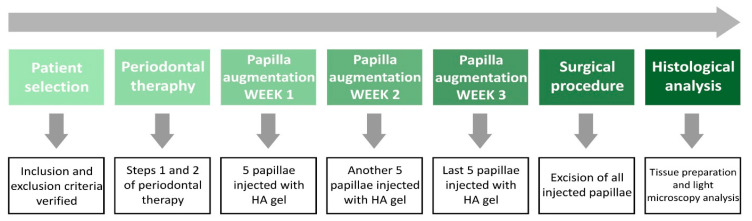
Time diagram.

This human histological study was designed as an experimental study with a duration of 4 weeks. Two patients contributed 15 papillae, which were injected once with HA gel (hyaDENT BG^®^). The papillae were injected in subsequent weeks for 3 weeks and during the 4th week, the papillae were removed as part of step 3 of surgical periodontal treatment [46]. At the end of the experiment, the last papillae were injected after 7 days of healing, while the first papillae were injected after 3 weeks of healing. Thus, papillae occurred at three-time points of healing after augmentation with HA gel. Immediately following the removal of the tissues, they were placed in a container containing 10% formalin and then transported to the oral pathology laboratory for histologic examination. The tissue samples were stained using the hematoxylin–eosin (H and E) stain, and the changes in the tissue were examined using a light microscope at different time intervals (Figure 1). The study received approval from the Research Ethics Committee of the Victor Babes University of Medicine and Pharmacy Timisoara. (approval no. 08/26.01.2024). The study was carried out in accordance with the guidelines for human medical experimentation provided in the Declaration of Helsinki. All participants provided written informed consent, allowing for dental procedures and collection of biological samples. The study was conducted between January 2024 and April 2024 and was registered in the ISRCTN Registry of Clinical Trials (ISRCTN12070271).

### 2.2. Patient Selection

Using the new classification system to diagnose periodontal and peri-implant diseases and conditions, systemically healthy, nonsmoking participants suffering from periodontitis stage III, grade B were screened for participation in the present research [47]. After completion of steps 1 and 2 of periodontal treatment, only the patients with the need for step 3 treatment due to not having reached the therapeutical target of ≤6 mm probing pocket depth (PPD) at re-evaluation after step 2 were recruited for participation from the Department of Periodontology, Victor Babes University of Medicine and Pharmacy Timisoara, Romania using the following inclusion and exclusion criteria [46].

*Inclusion criteria:*

- Nonsmoking adult patient.
- Presence of an uninterrupted frontal arch (maxillary or mandibular) of teeth affected by severe periodontitis.
- Presence of interproximal pockets deeper than 6 mm in at least 6 adjacent teeth.
- Class I, II, or III papillary recession, based on the classification of Nordland and Tarnow (1998) [48].
- Distance between the contact point and the crest of the alveolar bone equal to or greater than 5 mm.
- Keratinized tissue width of the entire area to be operated of at least 3 mm.
- Good oral hygiene—Plaque Index (PlI < 1 [49]).

*Exclusion criteria*:

- Patients who are diagnosed with systemic diseases, including hypertension, diabetes mellitus, or conditions that affect the effectiveness of periodontal therapy.
- Women who are currently pregnant or breastfeeding.
- Patients who use tobacco products.
- Patients with known allergies to hyaluronic acid or any excipients of the product used.
- Patients who are currently or have previously taken drugs that may increase the likelihood of gingival enlargement.
- Patients who are currently undergoing orthodontic treatment or have received orthodontic treatment within the past six months.
- Patients who have undergone traumatic dental hygiene procedures or periodontal surgeries within the past six months in the specific area of concern.

One experienced surgeon, a specialist in Periodontology (O-C.V.), performed all surgeries and augmentations using identical techniques for every papilla. All histological examinations were conducted by an investigator blinded (A.V.) to the duration of the healing periods.

### 2.3. Periodontal Therapy

The treatment of stage III grade B periodontitis patients was conducted according to the EFP S3 level clinical practice guideline. Patients received the first step of periodontal therapy, which is focused on controlling the supragingival biofilm [46]. The second step of therapy, which focuses on controlling the biofilm and calculus below the level of the gingiva (subgingival instrumentation), was carried out following the full-mouth disinfection protocol [50]. Under local anesthesia, ultrasonic instrumentation (EMS Piezon^®^ Master) using PerioSlim inserts (EMS, Nyon, Switzerland) and manual subgingival instrumentation with Gracey curettes (Hu-Friedy, Chicago, IL, USA), was performed. The procedure was completed using air polishing (PROPHYflex 3, KaVo KERR, West Collins Orange, CA, USA) [51]. OHI were reinforced after subgingival instrumentation. Four weeks following subgingival instrumentation, a follow-up appointment was arranged to evaluate the accomplishment of the therapy objectives and to verify the eligibility of the papillae sites for the study.

### 2.4. Papilla Aumentation Procedure

**Figure 2:**
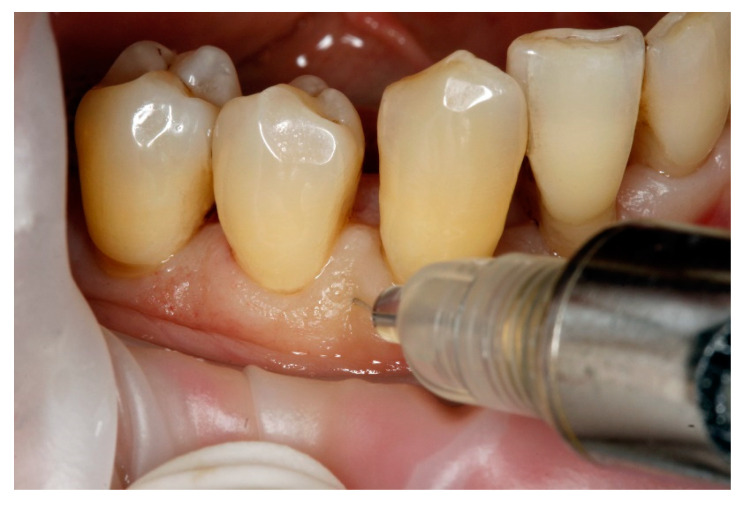
Clinical view of papilla injection with hyaluronic acid gel (hyaDENT BG^®^).

The papilla augmentation procedure was undertaken 4 weeks after completion of step 2 of therapy. A local anesthetic solution containing 4% articaine and 1:100,000 adrenaline was administered using a 30-gauge needle at the muco–gingival junction 5 min prior to the procedure. The HA gel was injected using a 30 G × 16/1300 needle inserted at a 45-degree angle, with the bevel facing towards the bone, 2–3 mm apical to the coronal tip of the chosen papilla. The product volume was customized to the specific needs of the patient until the adjacent tissue began to whiten, using about 0.2 mL of product [27]. Every week, two or three papillae were injected, resulting in papillae with varying healing times over a four-week period (Figure 2).

After injection, patients were instructed to avoid oral hygiene procedures for 24 h and resume them afterward with slight modifications: using a mouthwash with 0.20% chlorhexidine digluconate twice a day (Dentaton Intensivo, Dental Greenline, GHIMAS^®^, Bologna, Italy), refraining from using dental floss at the treatment sites; and brushing with a soft manual toothbrush coronal to the gingival margin. Patients were monitored weekly for 4 weeks.

### 2.5. Surgical Procedure

**Figure 3:**
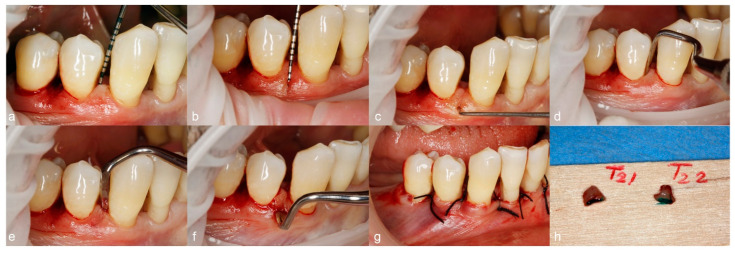
Illustration of intraoperative steps: papilla measurements (**a**–**c**); papilla incisions (**d**–**f**); sutured scalloped flaps (**g**); excised papillae with green staining on the base (**h**).

In the fourth week after the first injection, the patients underwent periodontal surgical therapy, and the injected vestibular papillae were surgically removed as part of step 3 of periodontal therapy [46]. A modification of the access flap was employed. Using an Orban knife, intrasulcular incisions ending in papilla split [52] into the interproximal areas were performed to separate the buccal from the oral hemipapilla at the level of the interdental gingival col, taking care to preserve the integrity of the buccal hemipapilla. At the base of each hemipapilla, horizontal incisions were made with the Orban knife, until the lateral blades of the knife reached the adjacent radicular surfaces. The buccal hemipapillae were lifted and removed using the triangular end of the Labanca periosteal elevator, while the oral hemipapillae were preserved for better flap closure (Figure 3).

The residual, almost linear buccal flap margin was trimmed to obtain a scalloped contour and new hemipapillae. Both buccal and oral flaps were then raised to full thickness, the root instrumentation was performed, bone deformities were removed using rotary instruments and chisels, and the flaps were secured in position with interrupted sutures made with non-resorbable 4-0 black silk suturing material (18″, C-3 Needle 13 mm, 3/8 Circle Premium Reverse Cut, PermaSharp, Hu Friedy, Chicago, IL, USA) (Figure 3).

Each excised buccal augmented papilla was stained with green ink on the base for better orientation (Figure 3). After the ink staining dried, the tissues were placed in a numbered container with 10% neutral-buffered formalin solution and sent for histological analysis.

### 2.6. Tissue Preparation

The histological processing and evaluation were performed in the Pathology Department of the Victor Babes University of Medicine and Pharmacy, Timisoara. The samples were fixed in 10% neutral buffered formalin for 24 h, afterward, they were oriented by identifying the oral epithelium and the ink staining on the base of the papilla and were perpendicularly sectioned along the longitudinal diameter. After the samples were embedded in paraffin wax, 4 µm serial sections were cut from each block at different levels, and subsequently stained with hematoxylin and eosin. Each slide comprised the lining epithelium and the underlining lamina propria. The slides were then scanned on a Leica Aperio AT2 slide scanner(Leica Biosystems, Deer Park, IL 60010, USA).

### 2.7. Outcome Measures

The following structures were evaluated during the microscopic analysis: the primary outcome was the amount of newly formed collagen fibers, seen as scattered thin bundles associated with the granulation tissue, as compared with compact, thick packages of pre-existing fibers. Secondary outcomes were (1) changes in the integrity of epithelium (or the presence of erosions/ulcerations on the epithelial aspects of the sections); (2) the presence and the characteristics (density) of the inflammatory infiltration; (3) the presence of granulomatous reactions; (4) the presence of interstitial edema; (5) the presence of the residual hyaluronic acid filler. All the outcomes were assessed at 1 week, 2 weeks, and 3 weeks post-injection with HA, and were recorded dichotomously on the specimens as the presence or absence of the parameter with the following scores: − (absence), + (low presence), ++ (moderate presence), +++ (high presence).

### 2.8. Statistical Analysis

To analyze the above-mentioned parameters, descriptive statistics methods to summarize and describe the main features of the dataset were used. The outcome scores (−, +, ++, +++) were interpreted as follows: 0 for the absence, 1 for low presence, 2 for moderate presence, and 3 for high presence (Table 1). Means, medians, and modes were calculated for all parameters and for all time points. The data were incorporated into observation tables and used to construct graphical representations of the changes.

## 3. Results

Two patients were included in this proof-of-concept histological human study, and 15 papillae in total were harvested during the surgical procedures. The study population consisted of two males ages 45 and 38 that contributed nine and six papillae, respectively.

During the experiment, no adverse reactions of any type were noted. Since the dimensional changes of the papillae resulting from the HA injection were of no interest to the present study, no morphological analysis was conducted.

**Figure 4:**
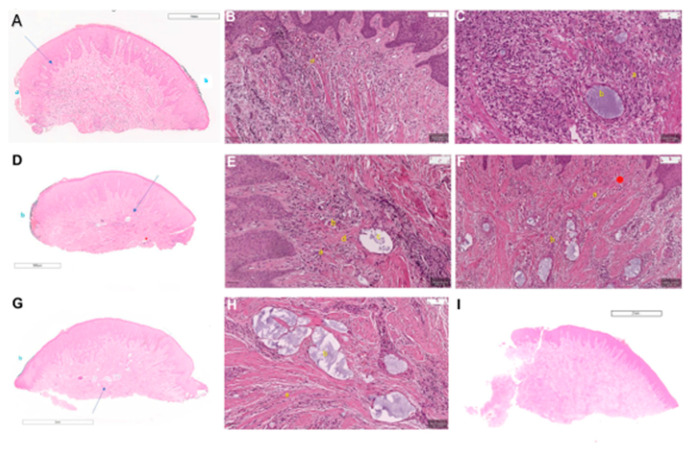
Histologic images of papillae from the three healing timepoints: (**A**) Overall view of a 4 µ thickness section of a sample harvested after the first week of healing: note the general rounded shape due to the lack of the papilla tip, and the epithelial erosion (a), green tint used to mark the papilla base that spread on the epithelium (b), higher magnification of section represented in (**B**) (arrow), sample T3.1, magnification 1×; (**B**) light microscopy of 4 µm section: cell-rich granulation tissue with dense inflammatory infiltrate and tissue edema (a), sample T3.1, scale bar = 30 µm, first week of healing; (**C**) light microscopy of 4 µm section: cell-rich granulation tissue with dense inflammatory infiltrate (a), hyaluronic acid pools (b), scale bar = 50 µm, first week of healing; (**D**) overall view of 4 µm section harvested after the second week of healing: green tint used to mark the papilla base that spread on the epithelium (b), higher magnification of section represented in (**E**) (arrow), sample T2.3, magnification 900×; (**E**) light microscopy of 4 µm section: subepithelial, maturing granulation tissue (a), decrease in the density of inflammatory infiltrate (b), hyaluronic acid pools (c), newly formed collagen fibers, thin and loosely packed (d), sample T2.3, scale bar = 50 µm, second week of healing; (**F**) light microscopy of 4 µm section: less interstitial edema (a), granulomatous reaction on hyaluronic acid pools surrounded by macrophages and multinucleated giant foreign body cells (b), scale bar = 100 µm, second week of healing; (**G**) overall view of 4 µm section of sample harvested after the third week of healing: green tint used to mark the papilla base that smeared on the epithelium (b), higher magnification of section represented in (**H**) (arrow), sample T1.3, magnification 2×; (**H**) light microscopy of 4 µm section: maturation of granulation tissue (a), hyaluronic acid lakes without granulomatous reactions (b), sample T1.3, scale bar = 50 µm, third week of healing; (**I**) overall view of a 4 µ thickness section of a non-injected sample: note the general aspect of an unaltered papilla used for comparison, sample T0, magnification 2×.

The sample harvesting during the surgery in step 3 of periodontal treatment resulted in a total of 15 papillae, with five samples for each time point. In none of the examined specimens were areas of necrosis identified, regardless of the time point. Because of the enlarged interdental spaces, the specimens did not have the characteristic pyramidal shape as observed in healthy individuals. Sections had typically flattened triangular shapes, with a large base of connective tissue at the horizontal incision level, and two rounded slopes with epithelial coverage. Most of the samples resulted in six sections each, but eight, four, or two sections per sample were also provided, depending on the state of the excised specimen. Sections of a non-injected control papilla belonging to one of the patients were used for comparison (Figure 4I).

### 3.1. Histological Changes at Week 1

The one-week post-procedure time-point showed tissue edema and granulation tissue development, along with a dense inflammatory infiltrate, as observed in Figure 4A,B. Blue-tinged HA filler pools were observed in the submucosa, with no evident granulomatous reaction developing around them (Figure 4C). The inflammatory infiltrate density was very high in the first week after the HA injection. The inflammatory cells were plasma cells with oval-shaped hyperchromatic nuclei and a large amount of cytoplasm, lymphocytes with round dark nuclei and small amounts of cytoplasm, and neutrophils with multilobulated nuclei. Fibroblasts with oval vesicular nuclei were present in a small amount in the first week post-injection. However, foreign body granulomas with giant cells could not be detected in the samples after the first week of healing. Interstitial edema with capillaries filled with erythrocytes was present and in high amounts on all samples after the first week, mostly around the HA pools (residual vesicles) that were also detected in each sample. In most samples, epithelial erosions (lack of the stratum corneum or the entire epithelium, without proliferation of the subjacent granulation tissue), and even epithelial discontinuities were noted. However, newly formed collagen could not be detected in the samples after the first week of healing after HA injection.

### 3.2. Histological Changes at 2 Weeks

At two weeks, interstitial edema and inflammatory infiltrate decreased in density, and newly laid collagen fibers that are thin and loosely packed became more evident, as observed in Figure 4D,E. Many of the HA pools presented a granulomatous reaction, characterized by the presence of macrophages and foreign-body multinucleated giant cells surrounding the filler material (Figure 4F). After the second week of healing, the inflammatory infiltrate density was also high, but not in the same amount as observed in the samples after one week. In two samples, foreign body granulomas with giant cells were detected, observed as large cells around the foreign material. Interstitial edema was present but was not as pronounced as after the first week. The HA gel started to fade away in the observed pools on some of the second-week samples; also, in half of the samples, epithelial erosions were present. A large number of fibroblasts could be observed, and newly formed collagen became visible on every sample as loosely packed thinner collagen bundles associated with areas of granulation tissue, after the second week of healing. The relatively thin bundles seemed to encapsulate the residual HA pools in most of the specimens. In some areas, the inflammatory infiltration seemed to be replaced by new collagen formation, while the inflammatory granulation tissue matures over time into scar tissue.

### 3.3. Histological Changes at 3 Weeks

The last time point was histologically characterized by a further decrease in the inflammatory and edema components. The granulomatous reaction around the HA filler pools was less obvious, being absent in most cases. A larger amount of new collagen was present, accompanied by a decrease in vascularity. The remaining blood vessels developed thicker vascular walls (Figure 4G,H).

The density of the inflammatory infiltration was not as pronounced after the third week of healing, as opposed to the first and second weeks. Foreign body granulomas with giant cells were observed in only one sample after the third week, and interstitial edema was not as pronounced in the third week after the HA injection. The HA pools were still observed in half of the samples, although the aspect of the material was no longer as compact as observed in the first week after injection. Epithelial erosions were almost non-detectable in the third-week samples. However, newly formed collagen was detected in each sample in high amounts as thinner bundles of collagen that are loosely packed when compared to the thicker and tightly packed mature pre-existing collagen bundles.

The mean, median, and mode of the primary outcome (new collagen fibers) and secondary outcomes (presence of HA, inflammatory infiltration, epithelial erosions, interstitial edema, and granulomatous reaction across) of three weeks can be summarized as follows in Table 2:

After the first week after HA injection, the newly formed collagen presented a mean, median, and mode of 0 that gradually rose to 1 (median and mode) after the second week, and to 2 (median and mode) after the third week, respectively. In the first week, HA had a mean median and mode of 1 that decreased to 1.4 (mean) in the second and third weeks after injection. All other variables also had a decreasing pattern throughout the weeks when it came to the mean, median, and mode calculated.

**Figure 5:**
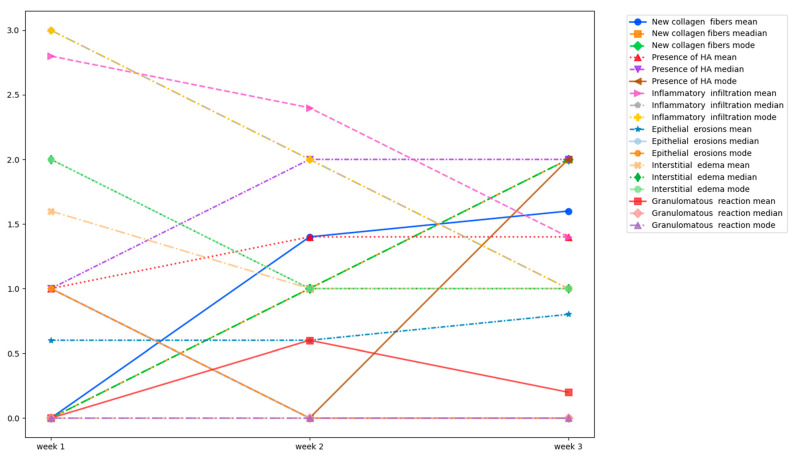
The mean, median, and mode of all parameters.

When analyzing the descriptive statistics and observing the composite graph, it appears that the new collagen fibers generally increased after the first and second weeks, the presence of HA decreased after the first week, and inflammatory infiltration decreased week by week along with the interstitial edema, which generally decreased after the first week (Figure 5).

## 4. Discussion

HA has been used in various treatment protocols to reduce inflammation and promote regeneration and wound healing [53,54,55,56].

There is ongoing debate regarding the clinical efficacy of HA filler injections in increasing the interdental papillary space, with some studies suggesting a beneficial augmentation effect [57,58,59,60], whereas current publications list potential adverse effects in addition to no apparent success in tissue augmentation procedures [61,62]. So far, such volumetric observations have not been sustained by histologic observations.

Reportedly, collagen synthesis is stimulated after HA filler injection, leading to an increase in collagen deposition surrounding the filler [30,63,64]. Its success in papilla augmentation is still controversial mainly because of the differences in HA formulations, injection protocols, host-related factors, and the standardization of the evaluation parameters [32].

Based on the information available to us, the present research is the very first histological study conducted on human subjects following a papilla augmentation procedure using injected HA gel. This study has been designed as a proof of concept, as the number of evaluated samples was limited because of the case design and of ethical considerations. However, this proof of concept may be of value for establishing a surgical protocol to reflect the histological modifications after augmentation injections in subsequent time points in the same individual and its limits for papillae sample harvesting in humans.

To date, there are only a few animal-model histological studies on this specific topic [32,44,45]. Histological evaluation of the gingival tissue was conducted in a pilot study 15 days after a 5% HA injection of an operculum. The study found that when comparing the results with the non-injected operculum on the opposite side, there were changes such as an increase in the number of epithelial cells and the presence of fibrous connective tissue with areas of concentrated fibroblast cells. These changes represent the initial stage of tissue enlargement in a clinical setting [65].

Injection with HA filler did not result in hypersensitivity or edema in a recent animal model study [45]. In our human study, no side effects like swelling, tenderness, local edema, or burning sensation were observed. Although the particular risks of injecting an HA filler have not yet been established, it has been hypothesized that partial obstruction of nearby blood vessels and external compression of the vascular circulation may be the source of side effects [66,67]. In our study, the location and the reduced volume of the augmented papillae make such adverse reactions unlikely.

### 4.1. New Collagen Formation

HA filler injections are reported to stimulate collagen synthesis by increasing the accumulation of collagen surrounding the filler [30,63,64]. An increase in the proliferation of human oral fibroblasts was observed in a recent in vitro study following HA treatment [41]. Our results are in accordance with these studies, as new collagen formation, starting from the second week after injection with HA, was observed. These newly formed collagen bundles appeared thinner and more loosely packed when compared to mature collagen fibers that are thicker and more tightly packed. Also, the quantity of collagen fibers is indirectly proportional with the quantity of inflammatory cells, over time less inflammatory cells and more collagen fibers are observed.

### 4.2. Residual HA Filler Presence

In our human histological study, the HA gel filler could be observed in most of the samples at every time point as an extracellular pale grayish-blue material contained in pools with diameters ranging between 100 and 300 microns and bordered by a fibrous capsule. Regarding human skin, the integration of HA dermal fillers demonstrated positive immune histological outcomes, with the gel-filled vesicles compacting beneath the epidermis [68]. The results of our study align with those of an animal-model study, which observed the presence of HA in a similar manner. However, in certain samples, the HA filler was extruded during the slicing protocol, likely due to the variation in hardness between teeth and gingival tissues [44]. A similar animal model study also found vesicles in the connective tissue after HA filler was injected into the interdental papilla [32]. A recent animal model study provided evidence of the HA filler being present in the connective tissue up until day 7 after the injection [45], results that are in accordance with our human histological study.

### 4.3. Presence and Characteristics of Inflammatory Infiltration

When inflammation occurs, many immune cells release various inflammatory mediators that induce vasodilatation and enhance blood circulation, producing redness and heat in the affected area [69]. A recent animal model research observed that following the injection of HA filler, a significant number of red blood cells were present in the connective tissue surrounding the filler [45]. A similar inflammatory infiltrate and interstitial edema were observed in our study in the specimens harvested after the first week of healing, as compared with the healthy papilla sample, where long rete pegs, thick collagen fibers, arranged in a parallel pattern, containing dense fibroblasts could be observed. At the end of the second week of healing, this infiltrate decreases in density, and the newly laid collagen becomes more evident. These findings are in accordance with previous human histological studies [56] where, after treatment with HA, angiogenesis was observed 10 days later.

### 4.4. Presence of Granulomatous Reactions

A granulomatous reaction could be observed in our study in almost half of the specimens, with a foreign body reaction, characterized by a granulomatous reaction, with macrophages and multinucleated giant cells surrounding the filler. This seems to contradict previous studies reporting that the HA filler does not stimulate the formation of granulomas, and does not have any impact on the metabolic activity of the fibroblasts, which implies that there are no adverse effects associated with using HA fillers in medical procedures, especially [68]. However, in our study, the local inflammatory reaction seems to coexist with the new formation of collagen fibers. This might be related to the bio-stimulant properties of the product, allowing a positive balance for connective tissue construction.

### 4.5. Interstitial Edema

Previous studies have suggested that local edema after oral HA injections could be caused by external vascular compression and partial occlusion of neighboring blood vessels [66,67]. Such interstitial edema could be observed throughout the healing time points in our research. Other animal studies reported that injection with HA filler did not result in hypersensitivity or edema [45]. Nevertheless, in our research, the intensity of the edema decreases from the first week to the third week after HA injection.

### 4.6. Epithelial Erosions

Epithelial erosions with unclear signification were more evident in our study after the first week of healing as compared to the last week. Possible explanations could be the trauma of the injection trajectory, and the fact that the epithelium in samples harvested after the first week could be more susceptible to handling trauma.

A possible limitation of the present human histological research is the limited number of patients and papillae, the reason for this being the very particular inclusion criteria. Another limitation of our study could be that only the early healing stages were investigated; however, the patients enrolled in this research were in need of step 3 periodontal treatment; prolonging this step to better investigate the HA presence in later stages would not have been in their best interest [46].

The lack of volumetric measurements after papilla augmentations could be considered a possible limitation of this research, as well. It is known that there are certain characteristics of the interdental space that must be present before augmentation in order to obtain the best volumetric results [13]. In patients with grade III, stage B periodontitis, these characteristics are rarely observed. Moreover, the cases included in this study did not fit the therapeutic purpose of filling the “black triangles”, both because the geometrical requirements for such an outcome (Nordland & Tarnow, 1998 “the rule of 5 mm” [13]) were not met, and because of ethical considerations. The lack of additional staining, such as Masson Trichrome staining, could also count as a possible limitation of our research; however, previous studies have discovered that HA does not survive after this staining process, so this staining has been considered not useful for our goal [44].

Future research should extend the period of follow-up for a better understanding of the hyaluronic acid role in the various stages of healing in the oral cavity. The correlation between volumetric clinical changes and histologic analysis could facilitate the development of therapies that will promote the augmentation of papillae lost from periodontal disease and the stability of results over time. In addition, assessing the impact of HA in individuals with systemic disorders that are more susceptible to impaired wound healing could have value for future treatments.

## 5. Conclusions

In terms of early wound healing, our human histologic research on the healing after injection with HA for papilla augmentation resulted in the observation that the formation of new collagen fibers occurs from the second week of healing; although a foreign body granulomatous reaction could be observed simultaneously with the presence of residual HA vesicles, this seems not to have impaired the fibroblast activity. Moreover, there is a variation among individuals in the process of HA consumption and its replacement with elements of connective tissue. This seems to depend on the amount of HA injected (patient-tailored, as per protocol), on the time passed since the injection, and on the local inflammatory status. 

## Figures and Tables

**Table 1:** Parameters scores.

Parameter	Week 1					Week 2					Week 3				
	Papilla 1	Papilla 2	Papilla 3	Papilla 4	Papilla 5	Papilla 6	Papilla 7	Papilla 8	Papilla 9	Papilla 10	Papilla 11	Papilla 12	Papilla 13	Papilla 14	Papilla 15
New collagen formation	0	0	0	0	0	2	1	2	1	1	2	2	2	1	1
HA presence	0	1	1	2	1	3	0	2	0	2	2	2	0	0	3
Inflammatory infiltration	3	3	2	3	3	2	3	2	3	2	1	1	1	3	1
Epithelial erosions	0	1	1	1	0	0	2	0	1	0	2	2	0	0	0
Interstitial edema	2	2	2	1	1	1	1	1	1	1	1	1	1	1	1
Granulomatous reaction	0	0	0	0	0	2	0	1	0	0	0	0	0	0	1

**Table 2:** The mean, median and mode were calculated for all parameters.

Parameter	Week 1			Week 2			Week 3		
	Mean	Median	Mode	Mean	Median	Mode	Mean	Median	Mode
New collagen fibers	0	0	0	1.4	1	1	1.6	2	2
Presence of HA	1	1	1	1.4	2	0 and 2	1.4	2	0 and 2
Inflammatory infiltration	2.8	3	3	2.4	2	2	1.4	1	1
Epithelial erosions	0.6	1	1	0.6	0	0	0.8	0	0
Interstitial edema	1.6	2	2	1	1	1	1	1	1
Granulomatous reaction	0	0	0	0.6	0	0	0.2	0	0

## Data Availability

The data presented in this study are available upon request from the corresponding author.
